# Cutting Pattern Identification for Coal Mining Shearer through a Swarm Intelligence–Based Variable Translation Wavelet Neural Network

**DOI:** 10.3390/s18020382

**Published:** 2018-01-29

**Authors:** Jing Xu, Zhongbin Wang, Chao Tan, Lei Si, Xinhua Liu

**Affiliations:** 1 School of Mechatronic Engineering, China University of Mining and Technology, No.1 Daxue Road, Xuzhou 221116, China; xujingcmee@cumt.edu.cn (J.X.); tccadcumt@126.com (C.T.); lei.si@cumt.edu.cn (L.S.); x.liu@soton.ac.uk (X.L.); 2 Institute of Sound and Vibration Research, University of Southampton, Highfield, Southampton SO17 1BJ, UK

**Keywords:** cutting pattern identification, sound signal, variable translation wavelet neural network, bat algorithm, ensemble empirical mode decomposition, disturbance coefficient

## Abstract

As a sound signal has the advantages of non-contacted measurement, compact structure, and low power consumption, it has resulted in much attention in many fields. In this paper, the sound signal of the coal mining shearer is analyzed to realize the accurate online cutting pattern identification and guarantee the safety quality of the working face. The original acoustic signal is first collected through an industrial microphone and decomposed by adaptive ensemble empirical mode decomposition (EEMD). A 13-dimensional set composed by the normalized energy of each level is extracted as the feature vector in the next step. Then, a swarm intelligence optimization algorithm inspired by bat foraging behavior is applied to determine key parameters of the traditional variable translation wavelet neural network (VTWNN). Moreover, a disturbance coefficient is introduced into the basic bat algorithm (BA) to overcome the disadvantage of easily falling into local extremum and limited exploration ability. The VTWNN optimized by the modified BA (VTWNN-MBA) is used as the cutting pattern recognizer. Finally, a simulation example, with an accuracy of 95.25%, and a series of comparisons are conducted to prove the effectiveness and superiority of the proposed method.

## 1. Introduction

Coal is an important fossil fuel and essential industry raw material, which always occupies almost 30% consumption of the primary energy in the whole. Shearer is the key equipment to guarantee the continuous, stable and safe running of underground coal mining [1,2]. Since the 1960s, scholars have paid much attention to the development of cutting pattern identification for the shearer, which focuses on recognizing whether the shearer is cutting coal, rock, or coal-gripping gangue. More than 20 kinds of cutting pattern identification methods have been researched, such as γ-ray detection [3], infrared detection [4], cutting temperature [5], vibration analysis [6], radar detection [7], etc. Among these, the online cutting sound signal, produced by the collision of the shearer cutting unit with the coal seam, has received much interest in recent years due to its non-contact measurement, simple structure, and low power. As a hot nondestructive testing method, the intelligent system according to the sound signal has gained a lot of attention and is widely applied in working condition monitoring [8], feature extraction [9], noise reduction [10], fault diagnosis [11,12,13], etc.

The acoustic-based cutting pattern recognition system actually extracts key information from the cutting sound signal and classifies them into several categories according to the characteristics. The wavelet transform (WT), developed from the Fourier transform (FT), is widely used in sound signal extracting due to its adaptive time-frequency window [14]. Moreover, with the rapid development of artificial neural network (ANN), many complex and nonlinear issues are well handled due to its self-adaption, self-organization, and real-time learning [15,16,17]. Unfortunately, the WT and ANN were completely separated in the early application, and the parameters in WT were fixed once selected. In 1992, the wavelet neural network (WNN), organically combining the WT and ANN, was proposed by Zhang et al. [18]. The transfer function in the hidden layer was the wavelet function in WNN instead of the traditional sigmoid function. The advantages of multi-scale and multi-resolution were also retained [19]. Therefore, the WNN has extensive applications in many fields. In [20], the rolling bearing fault diagnosis of a compressor system based on the WNN was proposed and verified. The Mexican hat wavelet function was used as the wavelet function, and the result indicated significant superiority over other neural networks. Turkoglu et al. designed an expert system for interpretation of the Doppler signals of heart valve diseases based on the WNN and achieved a recognition accuracy of 91% for 123 test samples [21]. A novel method for a noisy speech recognition model based on the integration of hidden Markov models and WNN was shown in [22]. Then, the WNN was applied in underwater acoustic communication in [23], and the acoustic channel simulations and pool experiments proved the method had faster convergence rate and convergence precision.

However, the basic WNN offered a fixed set of weight during the training process and was unable to obtain the characteristics of all input data. It was difficult to learn the input data deeply with fixed weight coefficients if the data were distributed in a wide domain [24]. Then, the variable translation wavelet neural network (VTWNN), where the translation parameters of the wavelets were variable depending on the network inputs, was developed to solve this problem [25,26]. Whether the WNN or the VTWNN, the training process was mainly based on the error back propagation (BP) algorithm. However, the BP algorithm easily falls into local extremum and has a low convergence rate, which causes extensive computation [27]. Thanks to the swarm intelligence optimization strategy, the training process for the WTNN ushered in a new development. Ling et al. presented a particle swarm optimization (PSO)-based VTWNN for modeling the development of fluid dispensing for electronic packaging [28]. In [29], a new intelligent PSO (IPSO) was used to optimize the parameters of VTWNN, and an affective design of mobile phones was applied to evaluate the effectiveness of the proposed method. The result showed that the proposed algorithm was significantly better than the other methods, with a 95% confidence level. In [30], the VTWNN was trained by the genetic algorithm (GA), and an application example on short-term daily electric load forecasting in Hong Kong verified the effectiveness of the proposed work.

Bat algorithm (BA) was a new meta-heuristic optimization algorithm proposed by Yang et al. in 2010 [31,32,33]. Bats expand their search scope by adjusting the intensity of the ultrasonic pulse and lock the location of prey through changing the emission frequency during the predation process. The bat-inspired optimization algorithm searched global optimal value by simulating bat foraging behavior. Recently, the BA was widely applied in data classification [34], scheduling [35], forecasting [36], artificial neural network model optimization [37], etc. BA was a powerful strategy and generated robust solutions on low-dimensional issues, but its performance weakened with dimension increases due to the limited exploration and exploitation abilities [38]. In order to improve the performance of the basic BA and avoid local optimization, some modified approaches were proposed to strengthen the local and global optimal value. In [39], a novel two-stage bat algorithm (TSBA) was designed to balance the relationship between exploration and exploitation using a trade-off strategy. Twenty-seven benchmark functions were utilized to illustrate advantages in terms of convergence rate and accuracy over other swarm intelligent optimization algorithms. Hasançebi et al. integrated an upper bound strategy with the basic bat-inspired algorithm (UBI) to realize the discrete sizing optimization of large-scale steel skeletal structures. The result showed the total number of structural analyses decreased 33.23% with respect to standard BA [40]. Besides, the bacterial foraging strategies were added into the BA to improve the positional accuracy of a wireless sensor network [41]. Many improvements focusing on the BA were elaborated in the past few years, but few researchers could balance both local extreme and iterative rate.

Bearing the above observations in mind, a cutting pattern identification system through the cutting sound signal was designed in this paper. The VTWNN was applied as the classifier, and the BA was used to determine the parameters of VTWNN instead of the traditional BP training process. Moreover, a disturbance coefficient was introduced into the basic BA to divide the bats into the native and explore group. The rest of this paper is organized as follows. In Section 2, the basic theory of the VTWNN and BA are described. Then, the modified BA (MBA) and the whole flow of proposed cutting pattern recognition scheme are illustrated in Section 3. Some simulations and comparisons according to a full-sized coal and rock seam are presented in Section 4 to validate the effectiveness and superiority of the proposed method. Finally, some conclusions and outlooks are summarized in Section 5.

## 2. Basic Theory 

### 2.1. Variable Translation Wavelet Neural Network

**Figure 1:**
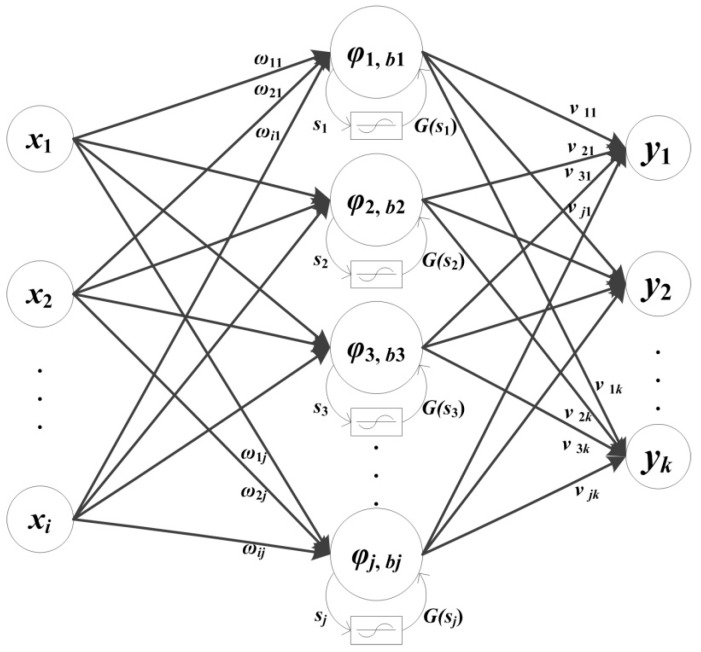
Structure of the variable translation wavelet neural network.

Wavelet neural network, proposed by Zhang et al. in 1992, is a kind of feedforward artificial neural network [18]. The WNN combines the multiscale wavelet transform and nonlinear neural network. The VTWNN was developed on the basis of the basic WNN with variable translation parameters of the wavelets according to the network input. The structure of the variable translation wavelet neural network is presented in Figure 1.

The VTWNN contains three layers: the input layer, hidden layer, and output layer [28,29]. The training process of VTWNN can be summarized as follows. Assume that each training sample is an *I*-dimensional vector *X* = (*x*_1_, *x*_2_, *x*_3_, …, *x_I_*)^T^. All input samples can be classified into *K* categories. Each input vector corresponds an output *Y* = (*y*_1_, *y*_2_, *y*_3_, …, *y_K_*)^T^. There are *J* nodes in the hidden layer by using wavelet function as the activator. The output of each hidden node is calculated as

(1) $$\varphi_{j,b_{j}}=\frac{1}{\sqrt{j}}\varphi (\frac{s_{j}-b_{j}}{j})$$

where *φ*(*x*) is the wavelet function, *s_j_* is the input of the hidden node, *j* and *b_j_* donate the wavelet scale and translation coefficient, respectively, and *j* = 1, 2, 3, …, *J*. The Mexican hat function is widely used as the mother wavelet function.

(2) $$\varphi (x)=(1-x^{2})\exp(-\frac{x^{2}}{2})$$

So the output of the hidden node can be presented as

(3) $$\varphi_{j,b_{j}}=\frac{1}{\sqrt{j}}(1-{\left(\frac{s_{j}-b_{j}}{j}\right)}^{2})\exp(-\frac{{\left(\frac{s_{j}-b_{j}}{j}\right)}^{2}}{2})$$

*s_j_* is calculated as follows:

(4) $$s_{j}=\sum_{i=1}^{I}x_{i}\cdot \omega_{ij}$$

where *x_i_* is the input vector of the network and *ω_ij_* is the weight coefficient between the input and the hidden layer. The translation coefficient *b_j_* is calculated as

(5) $$b_{j}=G(s_{j})=4j(\frac{2}{1+e^{-p_{j}\cdot s_{j}}}-1)$$

where *p_j_* is the tuned parameter and ranges generally from [0.3, 1.5]. Finally, the output node is given as

(6) $$y_{k}=\sum_{j=1}^{J}\varphi_{j,b_{j}}\cdot \nu_{jk}$$

where *k* = 1, 2, 3, …, *K* and *ν_jk_* donates the weight coefficient between the hidden and the output layer. The training process of the VTWNN is actually the determination of the value *ω_ij_*, *ν_jk_* and *p_j_* [26].

### 2.2. Bat Algorithm

Bat algorithm is a novel intelligent swarm optimization method first proposed by Yang et al. in 2010 [31]. The BA was a new kind of group evolution algorithm. The location of each bat represented a potential solution for the problem. The velocity and location of the bat were updated during the iteration to obtain the accurate place of the prey [42]. The iteration process of BA can be summarized as follows [32,33]:

Step 1.1: Parameters initialization. Initialize the population size of bat *P*, the range of pulse loudness *A*, the range of emission frequency *r*, the range of pulse frequency *f*, the attenuation coefficient of loudness *α*, the enhancement coefficient of emission frequency *γ*, and the iteration number *N*. Then, the position and velocity of *p*-th bat individual in a *D*-dimensional search space are randomly distributed in the feasible search space.

(7) $$\left\{ \begin{array}{l} x_{p,d}=x_{d\min}+(x_{d\max}-x_{d\min})\cdot rand(0,1)\\ v_{p,d}=\;v_{d\min}+(v_{d\max}-v_{d\min})\cdot rand(0,1) \end{array}\right.$$

where *p* = 1, 2, 3, …, *P*, *d* = 1, 2, 3, …, *D*, *x_p_*_,*d*_ is the coordinate index of *p*-th bat at *d*-th dimension, *v_p_*_,*d*_ is the corresponding speed, and *x*_min_, *x*_max_, *v*_min_ , and *v*_max_ are determined by the domain size of the problem of interest.

Step 1.2: Fitness comparison. The location of the bat is regarded as a potential solution of the optimization problem. The minimal fitness is selected as the best value.

(8) $$\begin{array}{l} fit=f(x_{p})\\ \left[bestfit,bestindex]=\min(fit\right) \end{array}$$

where *x_p_* is the location of the bat, *fit* is the fitness of the bat, *f*(·) is fitness function, *bestfit* is the minimal fitness of all bat individuals, and *bestindex* is the corresponding number.

Step 1.3: New location generation. The range of searching pulse frequency *f*, the flying speed, and location of each bat are updated as follows:

(9) $$\left\{ \begin{array}{l} f_{p}=f_{\min}+(f_{\max}-f_{\min})\cdot rand(0,1)\\ v_{p}^{n+1}=v_{p}^{n}+(x_{p}^{n}-x_{bestindext})\cdot f_{p}\\ x_{p}^{n+1}=x_{p}^{n}+v_{p}^{n+1} \end{array}\right.$$

where *x_bestindex_* is the corresponding location of the present best fitness and $v_{p}^{n}$ and $x_{p}^{n}$ are the flying velocity and location for *p*-th bat at *n*-th iteration, respectively.

Step 1.4: Random perturbation. If the generated value in the range of [0, 1] randomly is bigger than the pulse emission frequency *r*, then a perturbation is introduced for the bat on the basis of the present best solution.

(10) $$x_{new}=x_{bestindex}+\varepsilon A^{n}$$

where *ε* ∈ [−1, 1] is a random value and *A^n^* is the average loudness of all the bat individuals at this iteration step.

Step 1.5: Loudness and emission frequency variation. Generate a new random value in the range of [0, 1] and compare the fitness between the present and the new bat. If the following condition is satisfied, the new bat is adopted.

(11) $$rand(0,1)<A_{p}\;\&\;f(x_{p})<f(x_{bestindex})$$

Moreover, the loudness and emission frequency are updated in the next generation as follows:

(12) $$\left\{ \begin{array}{l} A_{p}^{n+1}=\alpha A_{p}^{n}\\ r_{p}^{n+1}=r_{p}^{o}(1-e^{-\gamma n}) \end{array}\right.$$

where *α* ∈ [−1, 1] is the attenuation coefficient and *γ* > 0.

Step 1.6: Global best evaluation. Calculate the fitness of each bat at the present generation and search the minimal fitness as the best, defined as *fitbest*. If *fitbest* is better than *bestfit*, than *bestfit* and the corresponding index number is replaced by the present optimal bat.

**Figure 2:**
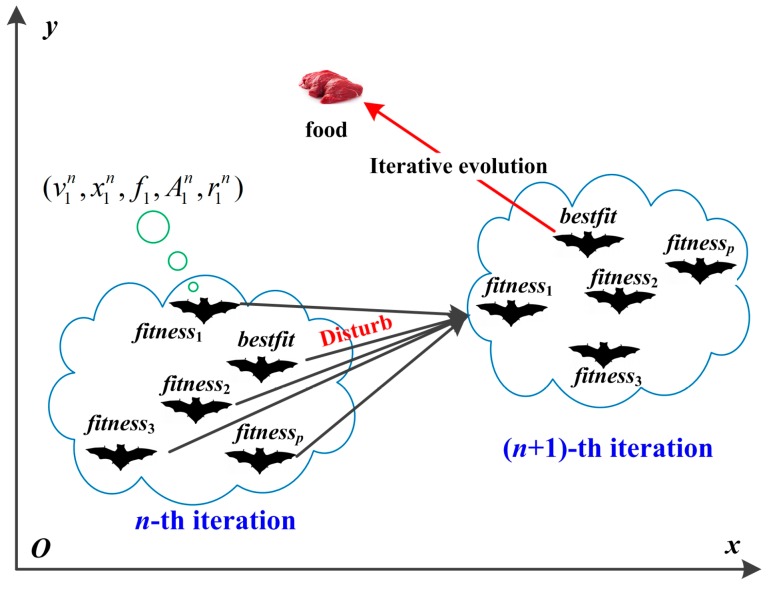
Foraging process of the bat swarm.

Step 1.7: Iteration termination. If the present iteration number *n* reaches the maximal *N* or the error satisfies the preset precision threshold value, the iteration process stops. Otherwise, steps 1.3 to 1.6 are repeated. The iteration process could be presented in Figure 2.

## 3. Algorithm Design

### 3.1. Modification of the Bat Algorithm

It can be seen from the BA searching process that the bats always fly toward the present best location during the generation of new individuals. Although the random perturbation is introduced in step 1.4, the moving distance decreases when the iteration increases. Once the bats fall into the local extremum in the later stage, it is difficult to jump the present area due to the lack of a powerful variation mechanism. Moreover, as the objective function is usually multimodal, the situation can become even more severe, especially for multiple parameters optimization. In this paper, the bat population is divided into two sections when the current best location remains unchanged for multiple iterations. Some of the bats (called native bats) continue searching for better position around the previous extremum, while the others (called explorer bats) are disrupted in a random way. Moreover, the bat individual is sorted into the above populations according to its fitness. The detailed iterative process of the modified bat algorithm (MBA) can be summarized as follows.

**Figure 3:**
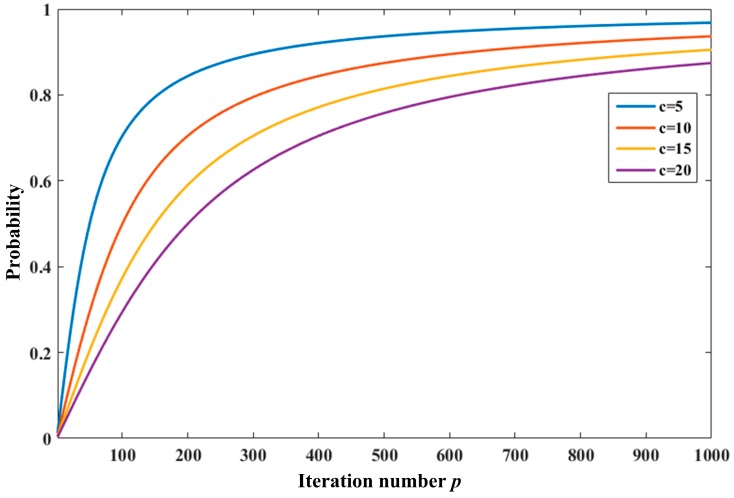
The probability distribution curve of a bat classified as the explorer.

A disturbance coefficient *c* is introduced into the MBA first. Assuming that the current best location is unchanged in the last *c* iterations, the bats are sorted in the order of smallest to largest in fitness. As the population size of bat is *P* and the bat optimization is actually searching for the minimal value, the bat with best fitness is numbered as 1 and the worst as *P*. The probability of one bat classified into the explorer population is defined as *g*.

(13) $$g_{p}=\frac{2}{\pi }\arctan(\frac{p}{10\cdot c})$$

where *p* = 1, 2, 3, …, *P*. The probability distribution curve with the change of *p* and *c* is shown in Figure 3.

It can be seen from the figure that probability increases with fitness, which means the bat with the best fitness has the smallest probability to be disturbed, while the one with the worst fitness has the biggest. For example, the first bat may be nregarded as the explorer with the probability of 0.64%, the 10-th of 5.71%, the 100-th with 50.00%, and 1000-th with 93.65% at *c* = 10. A small disturbance coefficient means a strength perturbation for the bat population. Here, the MBA can be regarded as the basic BA when *c* equals to the maximal iteration number *N*.

For the native population, all bats fly towards the current optimal position and their location and velocity are updated according to Equations (8)–(12). On the other hand, the explorer bats are flying to other area randomly as Equation (7). The two groups update independently, which indicates the natives and explorers fly to their own best locations, respectively. Once one of the populations gets a better fitness than the current global optimal, all bats fly toward the corresponding position. If the present global extremum remains unchanged for more than c iterations, the bats are resorted and divided into two groups, according to the above method. The pseudocode of the MBA is shown in Algorithm 1.

**Algorithm 1.** The pseudocode of modified bat algorithmInitialize *P*, *A*, *r*, *f*, *α*, *γ*, *N*, *c* and the objective function *f*(·). Initialize the position and velocity of each bat according to Equation (7). *n* = 0. Evaluate the fitness of each individual and find the best position *x_bestindex_*. while (*n* < *N*) if (*f*(*x_bestindex_*) remains unchanged more than *c* iterations)  Rank the bats according to their fitness and divide them into two populations.  Set *x_bestindex_* as the best native bat and generate new native bats as Equation (9).  if (rand(0, 1) > *r*)    Generate new native bat according to Equation (10).    if (the new bat satisfy Equation (11))      Accept the new native bat and update the loudness and emission frequency    end if  end if  Generate explorer bats randomly as Equation (7), calculate their fitness and find the best one *x_ebestindex_*.  Set *x_ebestindex_* as the best explorer bat and generate new explorer bats as Equation (9).  if (rand(0, 1) > *r*)    Generate new explorer bat according to Equation (10).    if (the new bat satisfy Equation (11))      Accept the new explorer bat and update the loudness and emission frequency    end if  end if  Evaluate the fitness of all bats and search the best one x*.  if (*f*(x*) is better than *f*(*x_ebestindex_*))    Accept x* as the optimal.  end if else  Set *x_bestindex_* as the best bat and generate new bats as Equation (9).  if (rand(0, 1) > *r*)     Generate new bat according to Equation (10).    if (the new bat satisfy Equation (11))      Accept the new bat and update the loudness and emission frequency    end if  end if  Evaluate the fitness of all bats and search the best one x*.  if (*f*(x*) is better than *f*(*x_ebestindex_*))    Accept x* as the optimal.  end if end if Search the current best bat. *n* = *n* + 1. end while Postprocess the results and visualization.

### 3.2. Flowchart of Cutting Pattern Method

In order to identify the cutting pattern of the coal mining shearer effectively, the cutting sound signal of the shearer is collected and analyzed in this paper. The cutting sound signal is first decomposed by adaptive ensemble empirical mode decomposition (EEMD). Then, the energy of each decomposition level is extracted as the feature vector. The VTWNN is applied as the recognizer. Moreover, the weight coefficients of the adjacent layers and the tuned parameters in VTWNN are trained by the MBA instead of the BP method in this paper. The flowchart of the proposed cutting pattern method through VTWNN optimized by MBA (VTWNN-MBA) can be summarized as follows.

Step 2.1: Decomposition of the initial cutting sound. The initial acoustic signal is segmented into *T* series, which can be divided into *T*_1_ training samples and *T*_2_ testing samples separately. In this paper, the initial sound was saved as .wav file. The sound signal collected directly from the field usually has the characteristic of strong nonlinearity, nonstationarity, and incontinuity. Therefore, it is of great importance to pretreat the signal through a suitable approach [43]. However, the most common time-frequency processing methods such as Fast Fourier Transform (FFT), Wavelet Transform (WT), and Wavelet Packet Transform (WPT) have difficulty satisfying the cutting sound signal. The FFT is restricted by the Dirichlet condition and Heisenberg uncertainty principle, which is inappropriate for a nonlinear and nonstationary signal. The WT and WPT have unavoidable defects for discontiguous signal as the wavelet basis and decomposition level are fixed once determined [44]. Empirical Mode Decomposition (EMD) was proposed by Huang et al. in 1998. EMD is an adaptive method to decompose any data into a set of IMFs, which become the basis of the data. As the basis is adaptive, the basis usually offers a physically meaningful representation of the underlying processes. In 2004, Ensemble Empirical Mode Decomposition was proposed by Wu et al. to deal with the mode mixing problem during EMD [45]. After 10 years of rapid development, EEMD is now widely applied in feature extraction [46], fault diagnosis [47], pattern recognition [48], etc. Assuming that the sound series can be decomposed into *M* intrinsic mode functions (IMFs) and a residue, the normalized energy of each IMF is calculated as the feature vector of the series.

Step 2.2: Parameters preset. Key parameters of VTWNN-MBA contain: the number of input layer in VTWNN *I*, the hidden layer *J* and the output layer *K*. So there exist *I*·*J* + *J*·*K* + *J* parameters in the VTWNN need to be optimized. The population size of bat *P*, the range of pulse loudness *A*, the range of emission frequency *r*, the range of pulse frequency *f*, the attenuation coefficient of loudness *α*, the enhancement coefficient of emission frequency *γ*, the iteration number *N* and the disturbance coefficient *c*. The original location of the *p*-th bat *ω_p_* = [*ω*_1_, *ω*_2_, *ω*_3_, …, *ω_D_*]^T^, *D* = *I*·*J* + *J*·*K* + *J*. The training samples are used to optimize the connection weight of the VTWNN-MBA, and the remaining testing series are applied to verify the cutting pattern recognition algorithm.

Step 2.3: Network optimization. *P* sets of weight coefficient solutions are generated, and the fitness value of each network is obtained according to Equation (14). When the recognition accuracy for the training samples reaches the minimal error, the network has its optimal structure. The iterative process is promoted according to MBA, and a series of new weight coefficient sets are produced in each circulation.

(14) $$fit=\frac{1}{T_{1}}\sum_{t=1}^{T_{1}}RMSE_{t}$$

(15) $$RMSE_{t}=\sqrt{\frac{1}{K}\sum_{k=1}^{K}{\left(Y_{t,k}-y_{t,k}\right)}^{2}}$$

where *T*_1_ is the number of training samples, *RMSE_t_* indicates the root mean square error for *t*-th acoustic series, *K* is the number of output layer, and *Y_t_*_,*k*_ and *y_t_*_,*k*_ donate the desired and actual value of *k*-th output node, respectively.

Step 2.4: Termination condition. If the iteration number reaches *N* or the fitness error less than *ξ*, terminate the iterative process; otherwise, continue the optimization.

**Figure 4:**
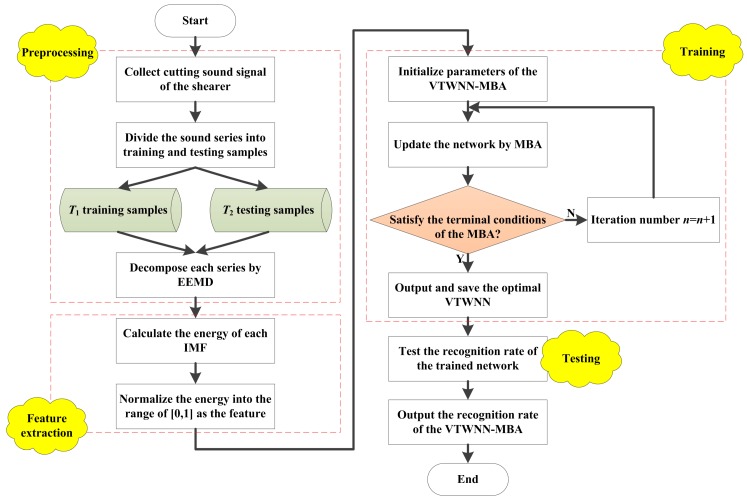
The flowchart of the proposed variable translation wavelet neural network modified bat algorithm (VTWNN-MBA).

Step 2.5: Network test. The VTWNN-MBA is trained by the *T*_2_ testing samples and the recognition rate is output. The flowchart of the VTWNN-MBA is shown in Figure 4.

## 4. Simulation and Analysis

In order to verify the validity and superiority of the proposed cutting pattern identification scheme, a simulation platform with different cutting patterns was put forward. A cutting acoustic signal with four working conditions was collected through an industrial microphone. Then, the original signal was normalized and decomposed successively. The key weight coefficient in the VTWNN-MBA was determined according to the training samples. The accuracy of the recognition network was validated by the testing samples. Some comparison and analysis were finally organized according to the simulation example.

### 4.1. Cutting Sound Acquisition and Pretreatment

**Figure 5:**
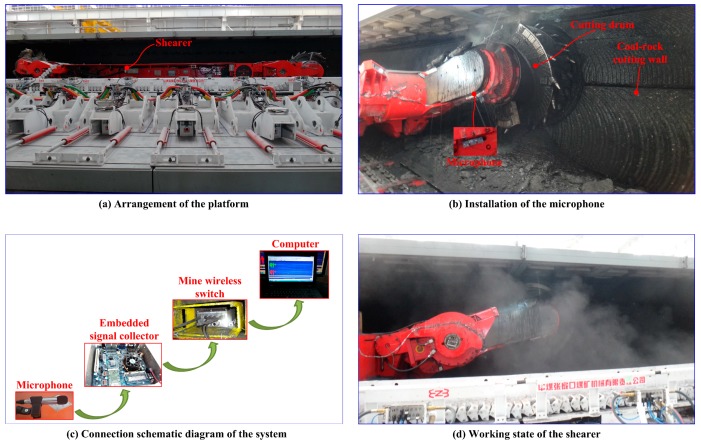
The experimental site.

**Figure 6:**
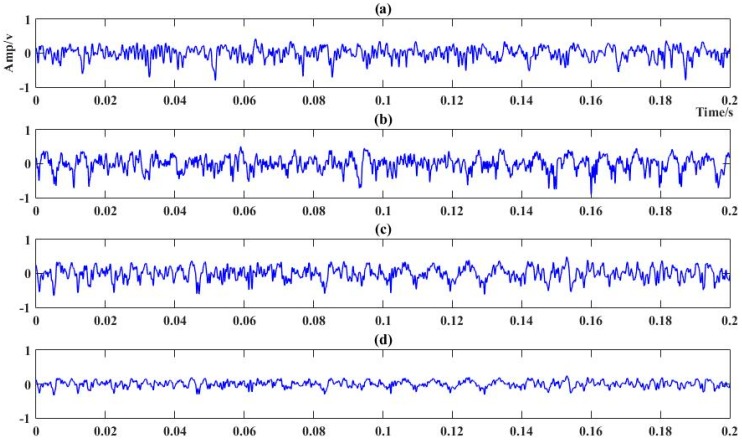
Cutting sound signal of the four cutting pattern. (**a**) Sound of coal seam with f2; (**b**) sound of coal seam with f3; (**c**) sound of coal seam gripping gangue; and (**d**) sound of no-load.

The original cutting acoustic signal was collected from the National Coal Mining Equipment Research and Experiment Center in Zhangjiakou, China. A full-sized coal and rock seam simulating the practical condition was built in the center. Then, an industrial microphone was installed on the coal mining shearer. The shearer type was an MG500/1130-WD (Ac traction shearer, Xi’an, Shanxi, China), the hauling speed of the shearer was 3 m/min, and the sampling frequency of the microphone was 44.1 kHz. The experimental site is shown in Figure 5. Four different kinds of sound corresponding to the shearer cutting coal seam with a Protodikonov hardness coefficient of f2 (S1), coal seam with a Protodikonov hardness coefficient of f3 (S2), coal seam gripping rock (S3), and no-load (S4) was recorded. Two hundred sound series were collected with a duration of 0.2 s for each cutting pattern. Half of them were regarded as the training samples, and the remaining were testing. The sound signal of four different kinds of cutting conditions are presented in Figure 6.

**Figure 7:**
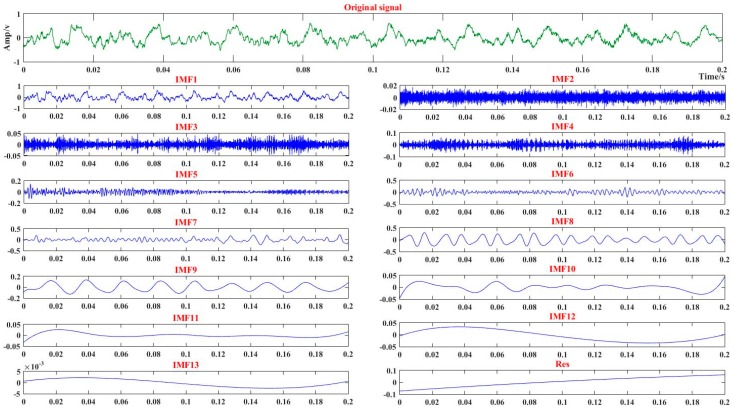
The ensemble empirical mode decomposition (EEMD) results of the cutting sound signal. The green line represents the original signal and the blue donates the IMF or Res of the EEMD result.

In order to extract key information from the original data, EEMD was applied to decompose the sound into a series of IMFs adaptively. The decomposition result are shown in Figure 7. The energy *E_nm_* of each IMF was calculated as the feature vector

(16) $$E_{nm}=\sum_{l=1}^{L}e_{nml}{}^{2}$$

where *E_nm_* represented the energy of *m*-th IMF for the *n*-th sample respectively, *L* was the length of the sample, and *e_nml_* donated the *l*-th element. Moreover, the normalization operation was then conducted to summarize the energy into the range of [0, 1]. For an arbitrary *x* ∈ [*x*_min_, *x*_max_], the normalization can be presented as follows:

(17) $$x_{N}=\frac{x-x_{\min}}{x_{\max}-x_{\min}}$$

where *x_N_* was the normalized value. Finally, the normalized energy of each IMF component was extracted as the feature vector as the input of the VTWNN-MBA. The feature vector of each sound series is shown in Table 1. It can be seen from the table that each acoustic fragment is presented as a 13-dementional vector from IMF1 to IMF13.

### 4.2. Training and Testing of the VTWNN-MBA

In order to recognize the cutting pattern of the coal mining shearer accurately, the VTWNN-MBA was trained and applied in this paper. The structure of the VTWNN was designed as follows: the number of the input layer *I* = 13, the hidden layer *J* = 6, and the output layer *K* = 4. So, there are 108 coefficients in the VTWNN that need to be determined. The key parameters of the modified bat algorithm were set as follows: the population size of bat *P* was 100, the range of pulse loudness *A* ∈ [0, 2], the range of emission frequency *r* ∈ [0, 1], the range of pulse frequency *f* ∈ [0, 2], the attenuation coefficient of loudness *α* = 0.9, the enhancement coefficient of emission frequency *γ* = 0.9, the iteration number *N* = 1000, and the disturbance coefficient *c* = 10. The acoustic samples were divided evenly between training and testing. The number of training series was 400, and the remaining 400 were testing ones. The desired output of the four different cutting pattern were S1 = [1, 0, 0, 0], S2 = [0, 1, 0, 0], S3 = [0, 0, 1, 0], and S4 = [0, 0, 0, 1] respectively. The average of root mean square error for all training samples was regarded as the fitness value.

**Figure 8:**
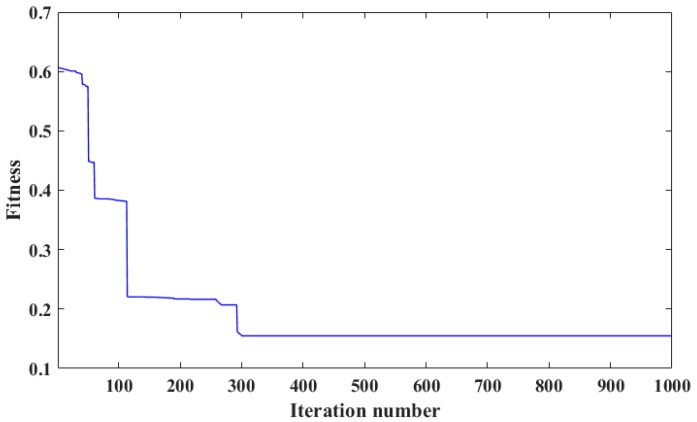
The iteration process of the VTWNN-MBA.

**Figure 9:**
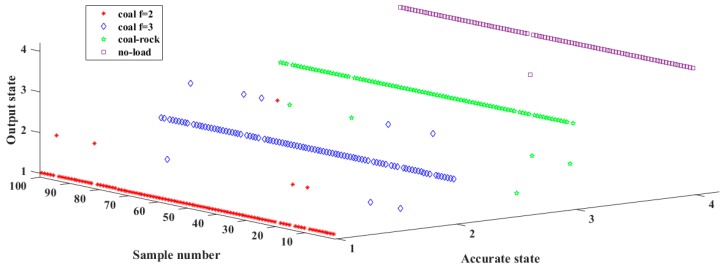
The recognition result of the testing samples.

The optimization was actually searching an appropriate 108-dimentional set to minimize the fitness. The iteration curve is shown in Figure 8. It can be seen in the iterative process that the final fitness was 0.154831. Then, the trained VTWNN was applied to identify the testing samples, and the recognition results are presented in Figure 9. Four hundred testing cutting acoustic series was input into the VTWNN, and 381 of them were recognized accurately. Specifically, four samples in S1 were misjudged into S2, and one was identified as S4. Three fragments in S2 were recognized as S1, and five were mistakenly classified into S3. Four series in S3 were sorted into S2, and one was into S1. One cutting sound sample in S4 moved to S3 by mistake. The recognition accuracy was defined as (*N*_1_/*N*_2_) × 100%, where *N*_1_ was the number of testing samples recognized correctly and *N*_2_ donated the total testing samples. So, the cutting pattern identification accuracy through the cutting sound signal by VTWNN-MBA was 95.25%. Through a deep analysis on the result, it can be seen that sound of cutting objects with similar hardness had little distinction, and those with evident differences could be identified precisely.

### 4.3. Comparison and Discussion

It can be seen from Equation (13) that the probability of one bat sorted into the explorer population was mainly determined by the disturbance coefficient *c*. In order to evaluate the impact of the disturbance coefficient, several contrast experiments were conducted, and the results are presented in Table 2. Different disturbance coefficient, such as *c* = 5, 10, 15, 25, 30, and 1000 (amount to BA) separately, and the corresponding iteration time for the 400 training samples, minimal fitness value and recognition rate were elaborated in the table. A small disturbance coefficient meant a strong disturbance during the optimization process. A bigger coefficient indicated weaker stimulus, and the bat group more easily fell into local extremum. When the bat population remained unchanged for several iterations, the explorer bats appeared immediately. It is shown in the table that the fitness value increased with the disturbance coefficient *c*, while the iteration time and recognition accuracy decreased. When *c* = 10, the recognition effect and the calculation time both reached a satisfactory value.

Moreover, seven other similar cutting pattern methods were also applied on the acoustic-based system to research the advantage of the proposed algorithm. In this paper, back propagation neural network (BPNN) [49], probabilistic neural network (PNN) [50], support vector machine (SVM) [51], the basic VTWNN, the VTWNN optimized by PSO, the VTWNN optimized by the GA, the VTWNN optimized by the original BA, and the proposed VTWNN-MBA were used to identify the cutting pattern, and the results are listed in Table 3. As can be seen from the table, the VTWNN-based methods had better fitness values and recognition accuracy for high dimensionality classification issues but cost more time due to the complex structure. Among the five VTWNN-based schemes, the swarm intelligence–based strategies had a better recognition rate and cost less time during the parameters optimization process. The basic VTWNN based on BP training had a simple structure. However, it had a low convergence speed and was easy trapping in local optimum. Further, the PSO-based method had the shortest calculation time due to its brief particle generation mechanism, and the GA was the most time-consuming among the four intelligence-based methods due to its complex crossover and mutation operations. The BA searched a balance between the above two methods. The VTWNN-MBA improved the identification rate 8.25% compared with the PSO algorithm and just cost 8.192333 s more time. On the other hand, it saved 19.10% time and achieved the same recognition accuracy when comparing with the GA-based method. As a whole, the proposed VTWNN-MBA had a good balance between the recognition rate and the calculation time.

## 5. Conclusions and Future Work

In order to identify the cutting pattern for the coal mining shearer, a novel scheme through the cutting sound signal based on VTWNN and an improved intelligent swarm algorithm was developed. The improved strategy on the basis of introducing a disturbance coefficient into the basic bat inspired algorithm was applied to enhance the ability to escape the present extremum. The intelligence optimization method was used for the parameters training process of the bat algorithm. To validate the effectiveness and advantages of the proposed method, a series of simulations was conducted, and some comparisons were analyzed. The simulation example and comparison results show that the acoustic-based cutting pattern identification method can accurately distinguish the cutting pattern, and the proposed approach preceded other algorithms.

However, there are also some limitations in this method that can be summarized as follows: (1) the disturbance coefficient in MBA is selected mainly through vast computer simulations. The lacking of rigorous derivation will increase the uncertainty of the system. (2) The proposed VTWNN-MBA is still time-consuming, so the execution efficiency of the code needs to be improved. In future studies, the authors plan to implement some improvements on the proposed method. These may include a strict mechanism to select an appropriate disturbance coefficient in MBA and a shorter calculation time of the algorithm code to realize online recognition.

## Figures and Tables

**Table 1:** Feature vector of the acoustic series.

Sample Number	Feature Vector
1	[0.493820, 0.018635, 0.002433, 0.003701, 0.001007, 0.000861, 0.000946, 0.000362, 0.000330, 0.000204, 0.000200, 0.000091, 0.000046]
2	[0.744507, 0.190640, 0.001730, 0.003545, 0.000902, 0.000844, 0.000783, 0.000187, 0.000305, 0.000197, 0.000167, 0.000080, 0.000140]
3	[0.700600, 0.081532, 0.001633, 0.004464, 0.000536, 0.000669, 0.000517, 0.000216, 0.000437, 0.000244, 0.000163, 0.000132, 0.000025]
4	[0.363571, 0.066428, 0.003079, 0.004894, 0.000692, 0.000852, 0.000895, 0.000415, 0.000399, 0.000256, 0.000155, 0.000107, 0.000003]
5	[0.480629, 0.035871, 0.009238, 0.014017, 0.001057, 0.001220, 0.003743, 0.000455, 0.000014, 0.000180, 0.000214, 0.000052, 0.000125]
6	[0.767436, 0.023610, 0.002480, 0.002233, 0.000964, 0.000818, 0.000401, 0.000157, 0.003202, 0.000255, 0.000136, 0.000823, 0.000227]
…	
799	[0.772048, 0.016429, 0.021885, 0.009308, 0.002668, 0.000636, 0.000302, 0.004158, 0.000097, 0.000159, 0.001217, 0.000137, 0.000038]
800	[0.268025, 0.015486, 0.001868, 0.007008, 0.000349, 0.001086, 0.001178, 0.000568, 0.000233, 0.000230, 0.000118, 0.000140, 0.000049]

**Table 2:** Comparisons between the different disturbance coefficients.

Disturbance Coefficient	Iteration Time (s)	Fitness Value	Recognition Accuracy
5	65.962150	0.150311	95.25%
10	64.201883	0.154831	95.25%
15	62.193844	0.163709	94.50%
25	62.001930	0.180094	94.25%
30	61.003760	0.183762	92.50%
1000	60.227091	0.201358	91.50%

**Table 3:** Comparisons between the different cutting pattern identification methods.

Compared Methods	Iteration Time (s)	Fitness Value	Recognition Accuracy
BPNN	82.675028	0.330370	78.75%
PNN	89.002130	0.310938	82.50%
SVM	83.309544	0.311052	82.50%
VTWNN	92.395211	0.310279	84.75%
VTWNN-PSO	56.009550	0.229624	87%
VTWNN-GA	79.362199	0.160962	95.25%
VTWNN-BA	60.227091	0.201358	91.50%
VTWNN-MBA	64.201883	0.154831	95.25%
